# Supplementary material for: Efficient and sustainable water electrolysis achieved by excess electron reservoir enabling charge replenishment to catalysts
Source: Nat Commun. 2023 Sep 5;14:5402. doi: 10.1038/s41467-023-41102-2 (PMC10480199; doi:10.1038/s41467-023-41102-2)
Supplement: Supplementary file 1 — Supplementary Information [file 41467_2023_41102_MOESM1_ESM.pdf]

Supplementary Information

Efficient and Sustainable Water Electrolysis Achieved by Excess Electron Reservoir Enabling Charge Replenishment to Catalysts

Gyu Rac Lee[1+], Jun Kim[2+], Doosun Hong[3+], Ye Ji Kim[1,4], Hanhwi Jang[1], Hyeuk Jin Han[5], Chang-Kyu Hwang[6], Donghun Kim[3*], Jin Young Kim[2*] and Yeon Sik Jung[1*]

[1]Department of Materials Science and Engineering, Korea Advanced Institute of Science and Technology, 291 Daehak-ro, Yuseong-gu, Daejeon 34141, Republic of Korea
[2]Hydrogen·Fuel Cell Research Center, Korea Institute of Science and Technology, 14-gil 5, Hwarang-ro, Seongbuk-gu, Seoul 02792, Republic of Korea
[3]Computational Science Research Center, Korea Institute of Science and Technology, 14-gil 5, Hwarang-ro, Seongbuk-gu, Seoul 02792, Republic of Korea
[4]Department of Materials Science and Engineering, Massachusetts Institute of Technology, Cambridge, Massachusetts, 02139 United States
[5]Department of Environment and Energy Engineering, Sungshin Women's University, 55, Dobong-ro 76ga-gil, Gangbuk-gu, Seoul 01133, Republic of Korea
[6]Materials Architecturing Research Center, Korea Institute of Science and Technology (KIST), 14-gil 5, Hwarang-ro, Seongbuk-gu, Seoul 02792, Republic of Korea

Table of Contents

Supplementary Figures **3**

Supplementary Tables **34**

Supplementary References **36**

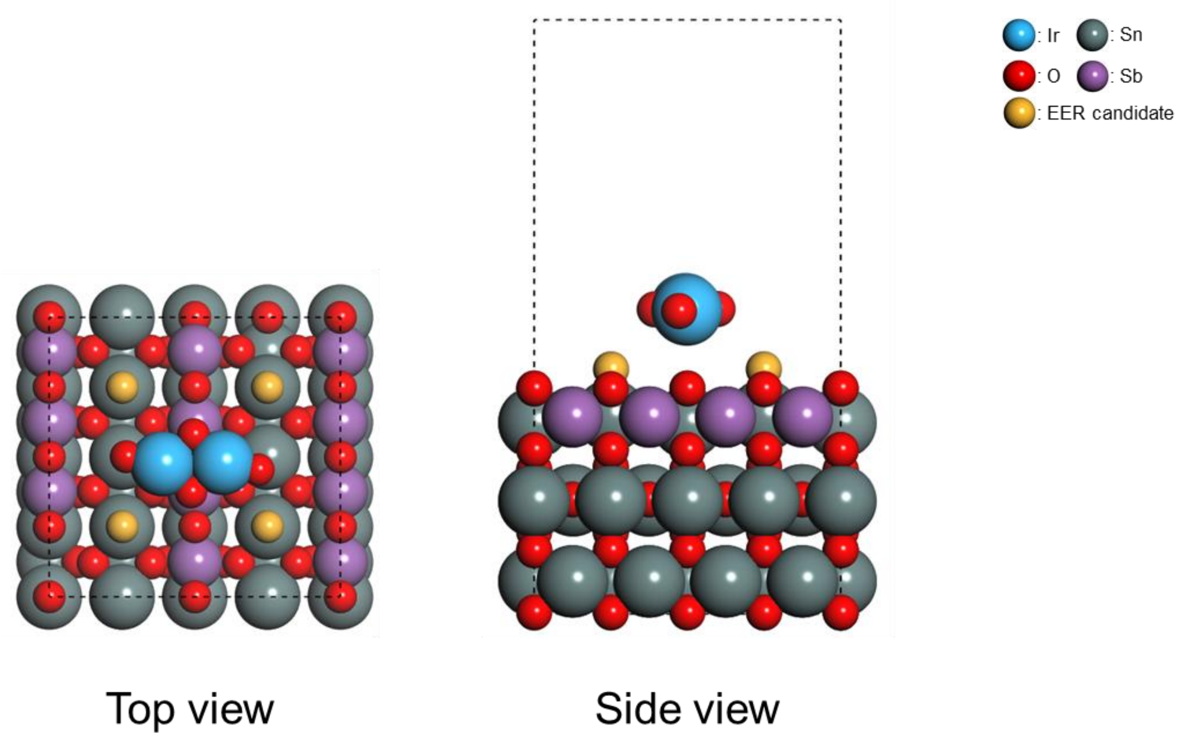

Supplementary Fig. 1. Computational screening: DFT modeling of IrO_x with the EER-contained ATO support.

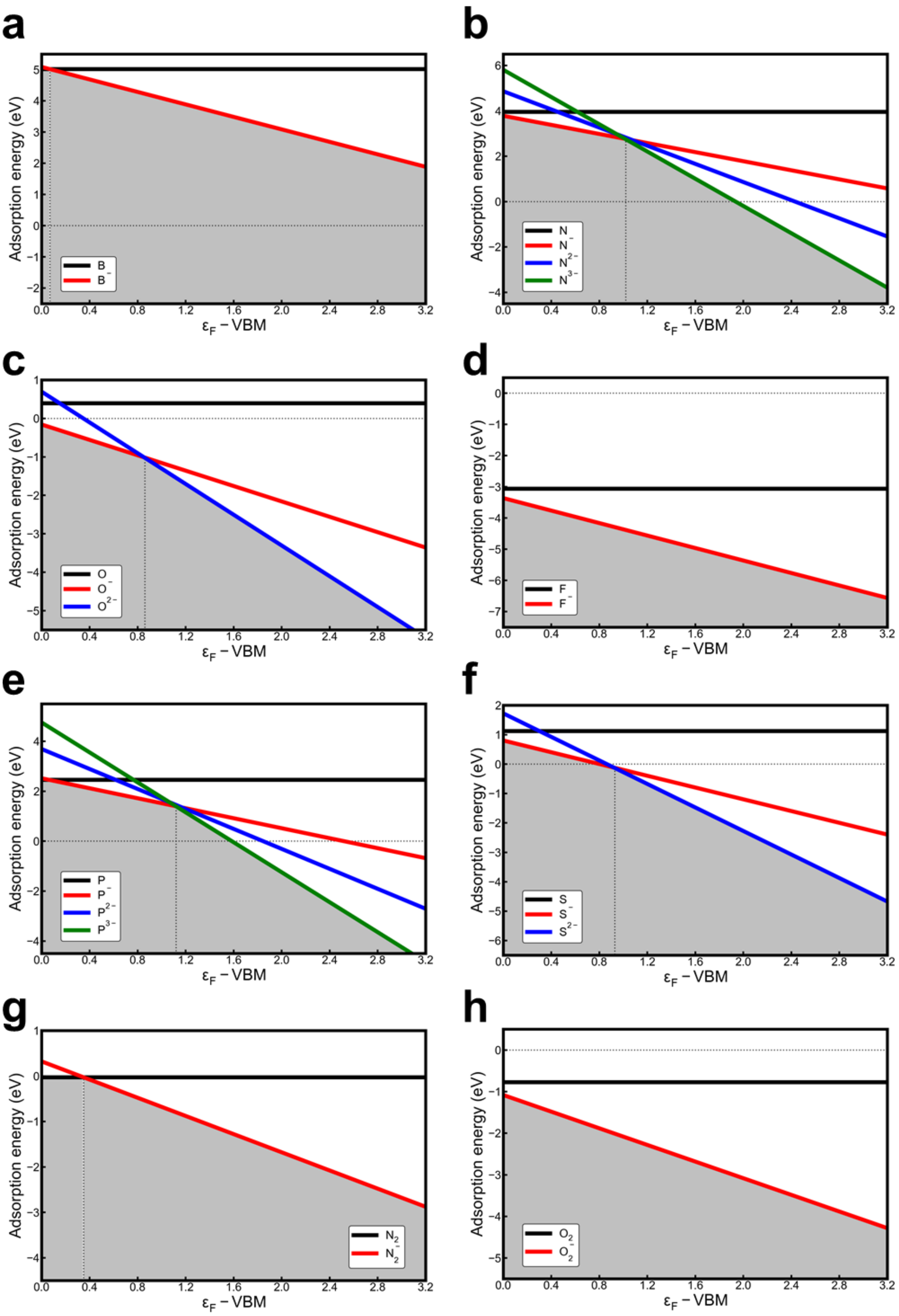

Supplementary Fig. 2. Computational screening: adsorption energy versus Fermi level of **a** B and B^-, **b** N and N anions, **c** O and O anions **d** F and F- **e** P and P anions **f** S and S anions, **g** N_2 and N_2^-, **h** O_2 and O_2^-. The gray area indicates the most stable form of each species.

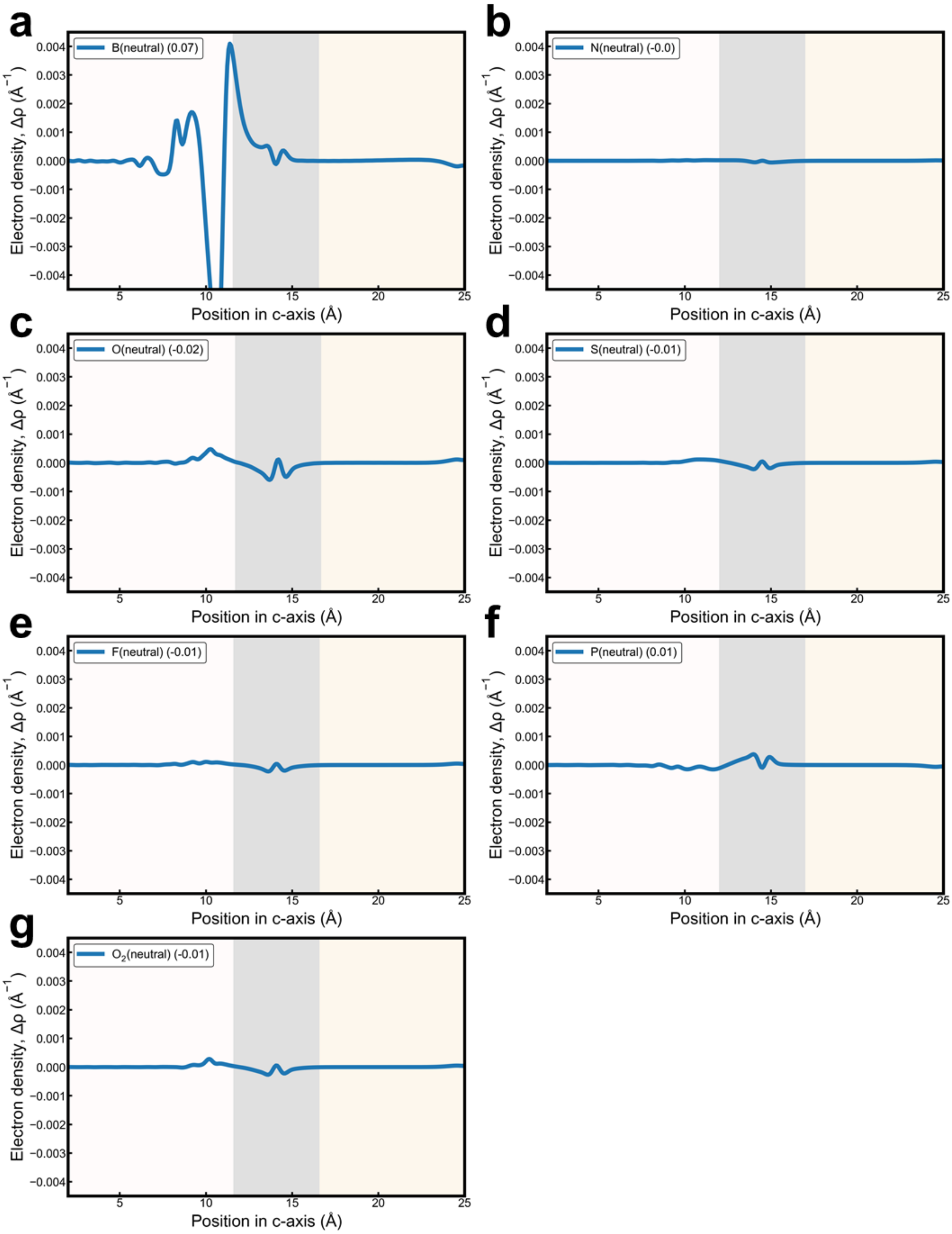

Supplementary Fig. 3. Computational screening: Neutral EER candidate-induced electron transfer plotted along the c-axis. The gray area indicates the IrO_2 cluster region and the numbers in parentheses are the amount of electron transfer to the IrO_2 cluster by **a** B **b** N **c** O **d** S **e** F **f** P **g** O_2.

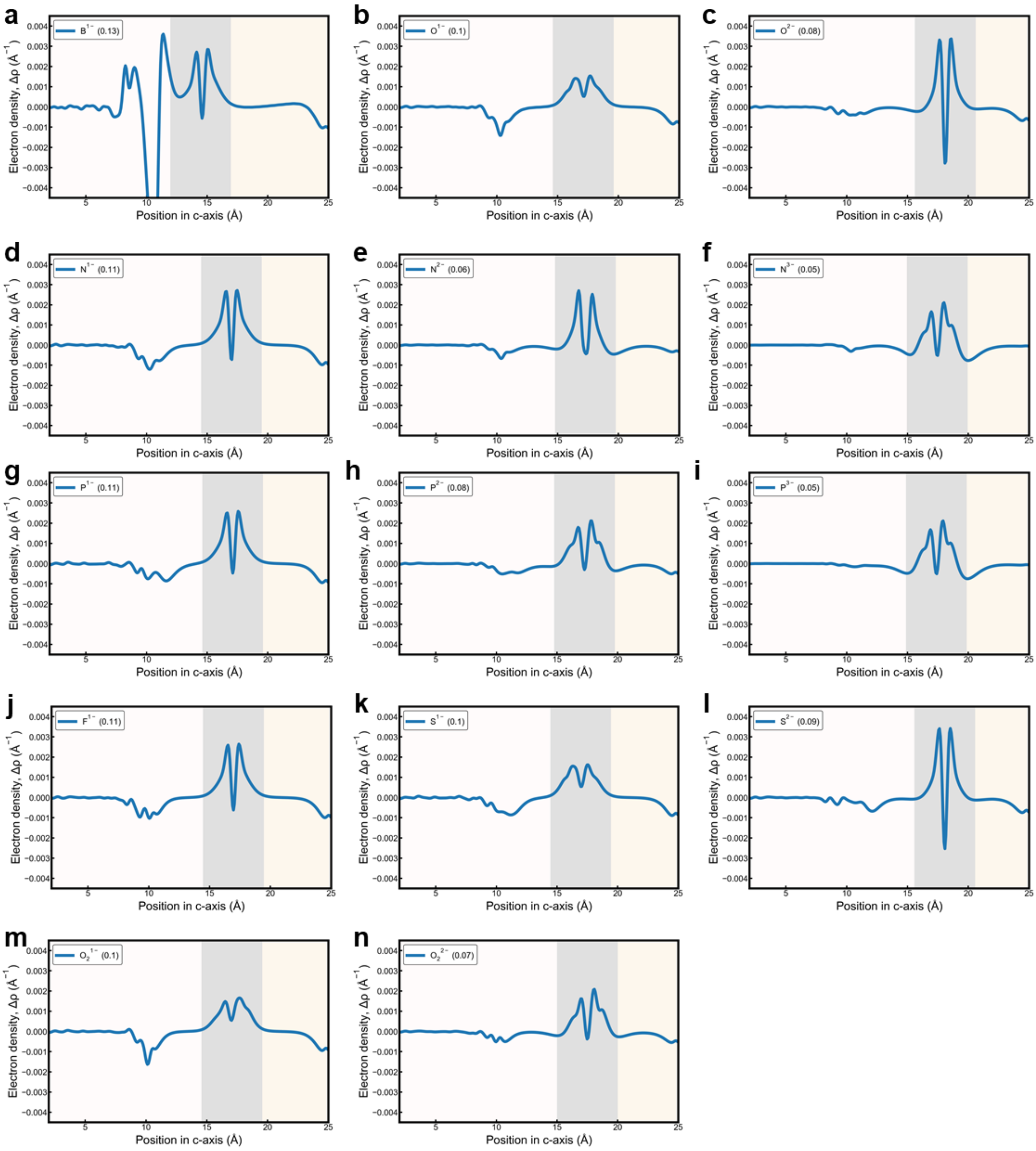

Supplementary Fig. 4. Computational screening: Charged EER candidate-induced electron transfer plotted along the c-axis. The gray area indicates the IrO_2 cluster region and the numbers in parentheses are the amount of electron transfer to the IrO_2 cluster by **a** B^-, **b, c** O anions, **d, e, f** N anions, **g, h, i** P anions, **j** F^-, **k, l** S anions, **m, n** O_2 anions.

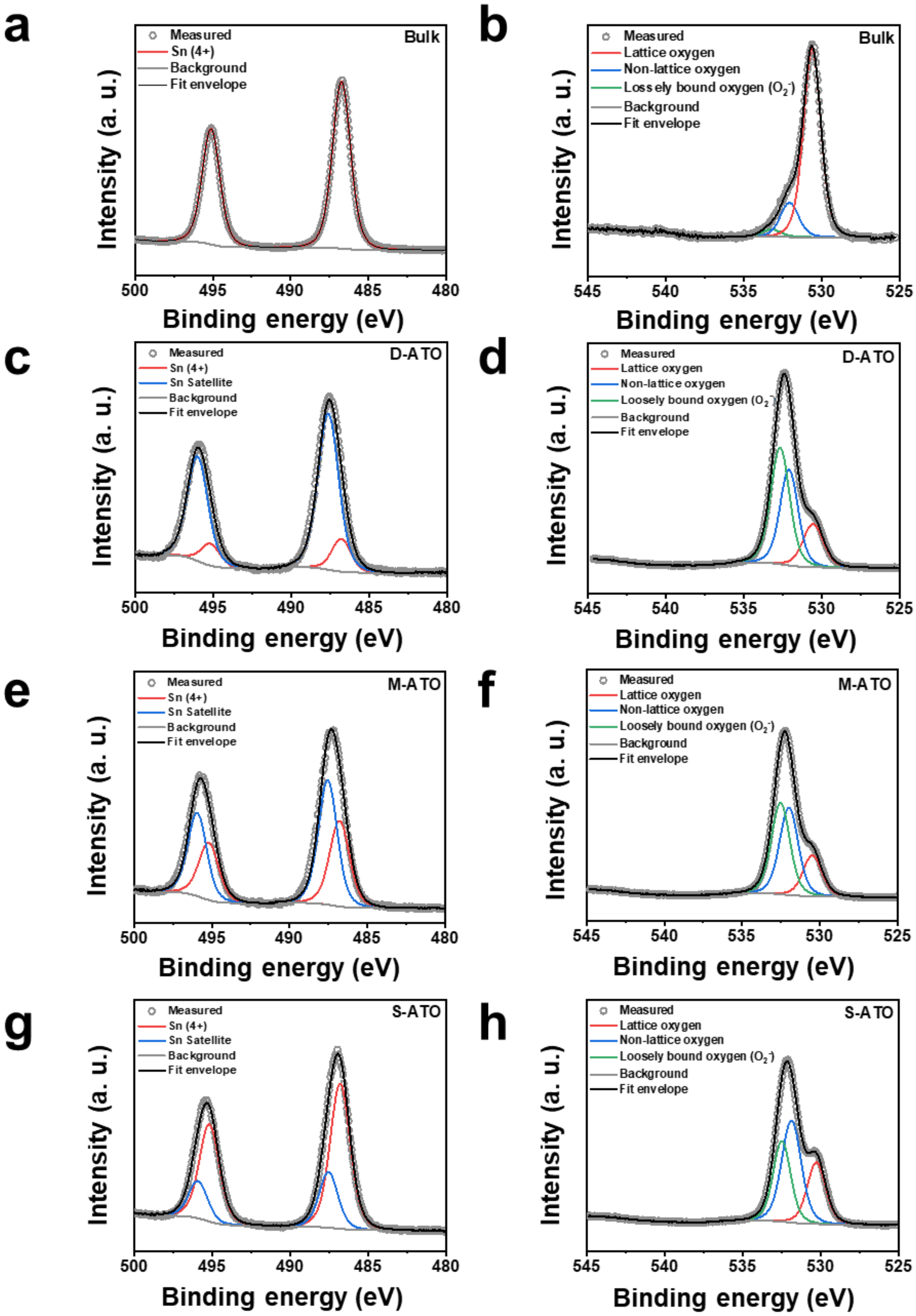

Supplementary Fig. 5. X-ray photoelectron spectroscopy (XPS) spectra of bulk and EER-contained ATO nanowire arrays with varying density of EER. The binding energies of Sn 3d spectra (**a, c, e, g**) and O 1s spectra (**b, d, f, h**) of each sample, respectively. Note that a. u. represents arbitrary units.

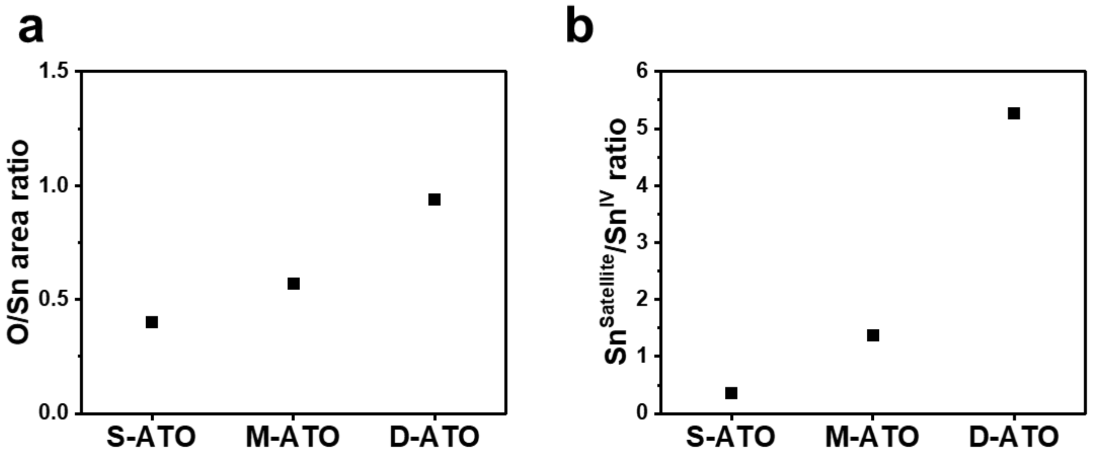

Supplementary Fig. 6. Criteria for the definitions of S-ATO, M-ATO, and D-ATO. a O/Sn area ratio and **b** $Sn^{Satellite}/Sn^{IV}$ ratio of the ATO with varying EER content. The O/Sn ratio was analyzed using the peak intensity of the O 1s and Sn 3d peaks. The $Sn^{Satellite}/Sn^{IV}$ ratio was calculated using the peak intensity of the Sn 3d peaks deconvoluted into $Sn^{Satellite}$ and Sn^{IV}.

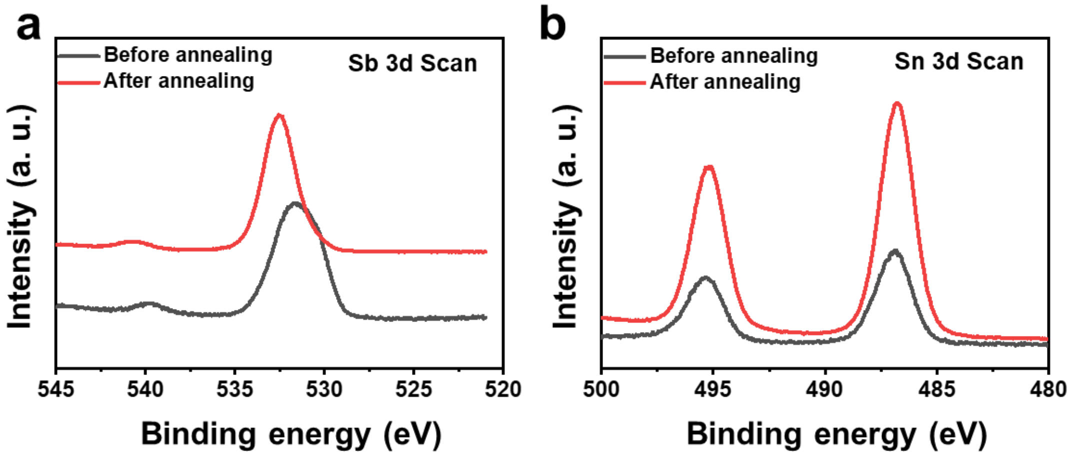

Supplementary Fig. 7. X-ray photoelectron spectroscopy (XPS) spectra of **a** Sb 3d and **b** Sn 3d of dense EER ATO (D-ATO) nanostructure before and after annealing to confirm the doping of Sb to the SnO_2 lattice. Note that a. u. represents arbitrary units.

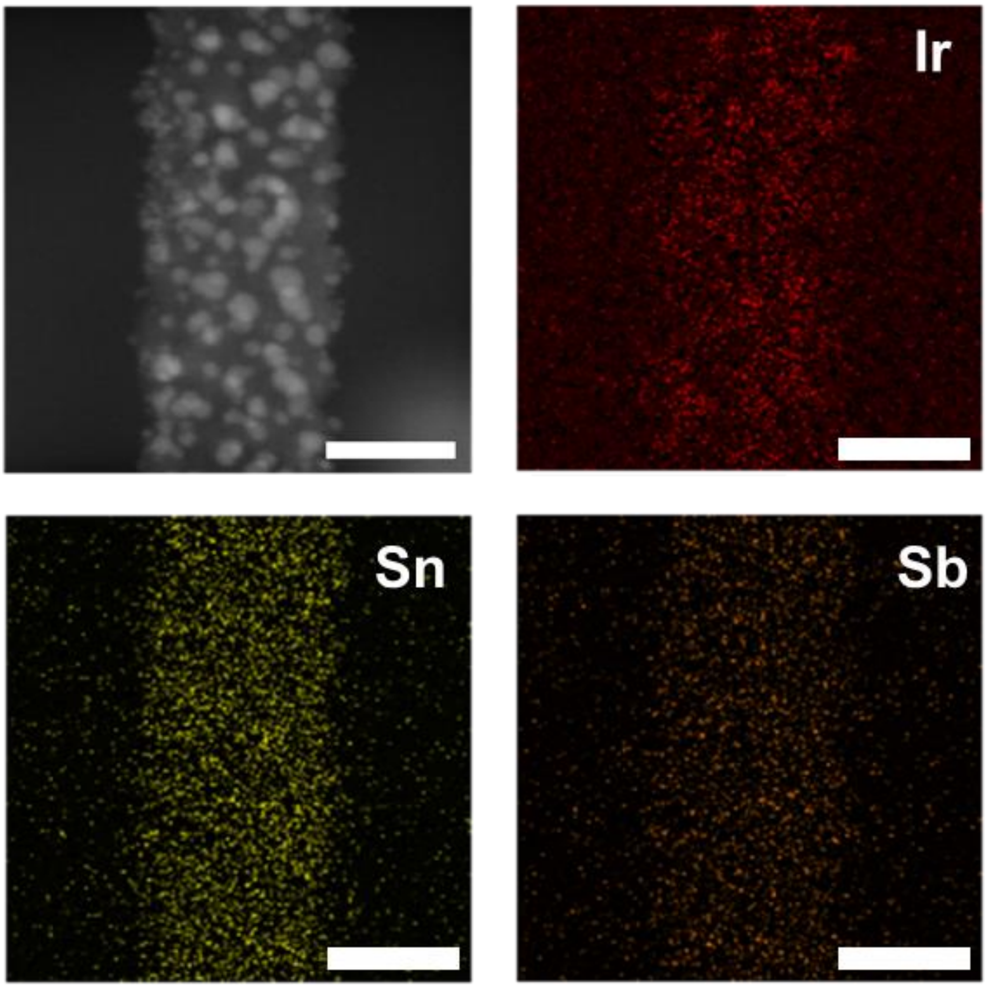

Supplementary Fig. 8. Scanning transmission electron microscopy (STEM) image and energy dispersive spectroscopy (EDS) mapping images of Ir/D-ATO with 50 nm scale bars.

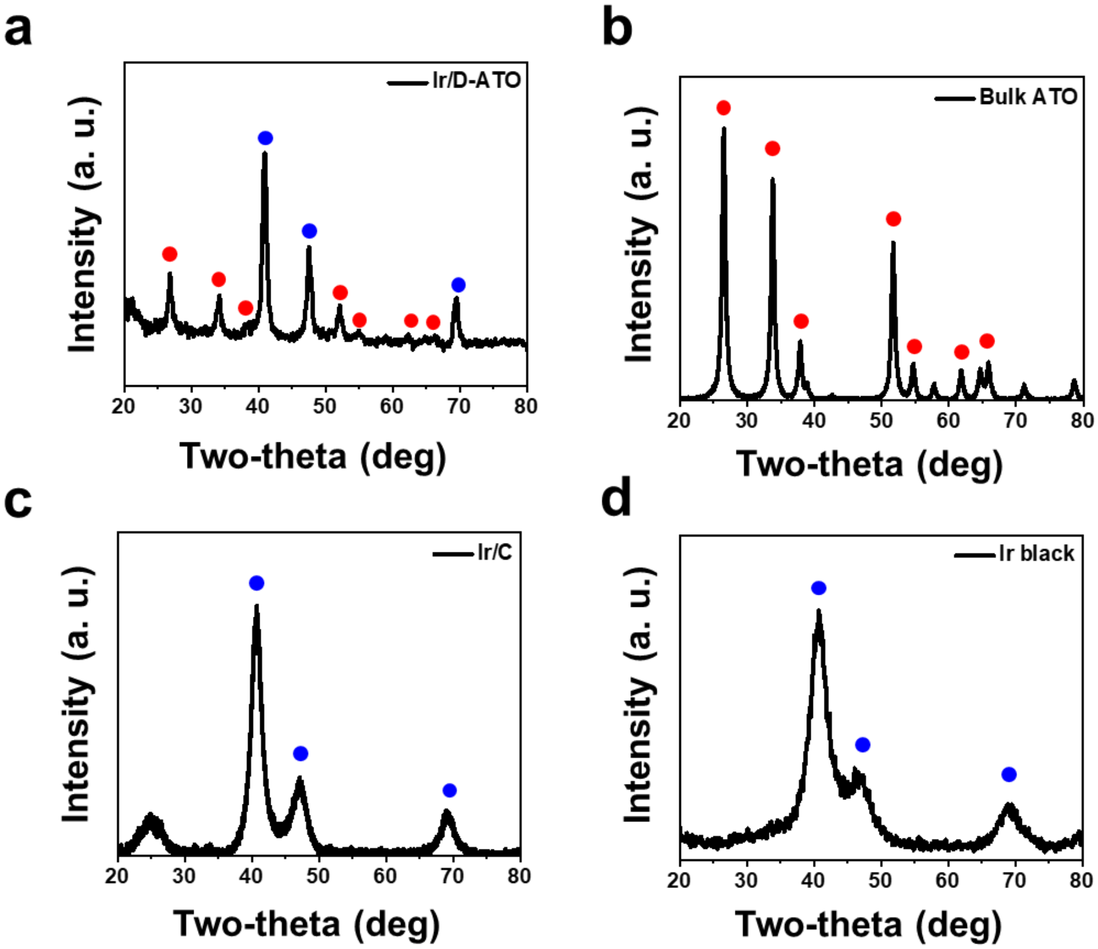

Supplementary Fig. 9. X-ray diffraction (XRD) spectra of the **a** Ir/D-ATO, **b** bulk ATO powder, **c** Ir/C, and **d** Ir black using 2-theta scan from 20° to 80°. Blue dots indicate the dominant facets within the crystal structure of iridium metal and red dots indicate the dominant facets within the crystal structure of antimony doped tin oxide. Note that a. u. represents arbitrary units.

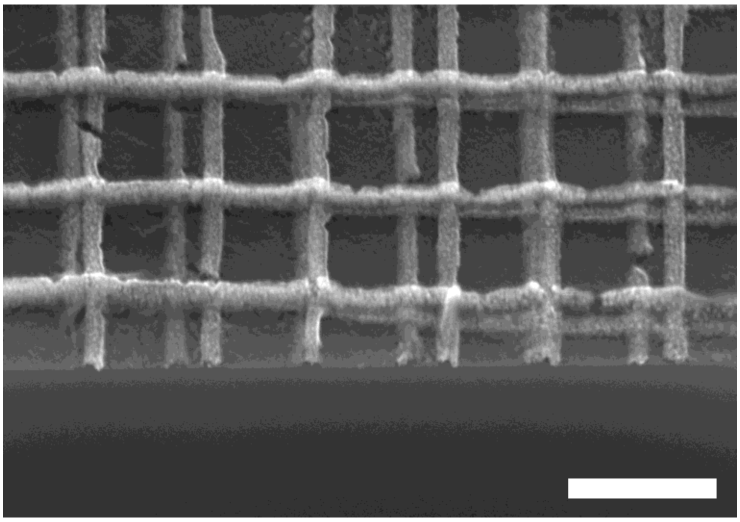

Supplementary Fig. 10. Scanning electron microscopy (SEM) image of the final morphology of Ir/D-ATO with 500 nm scale bar.

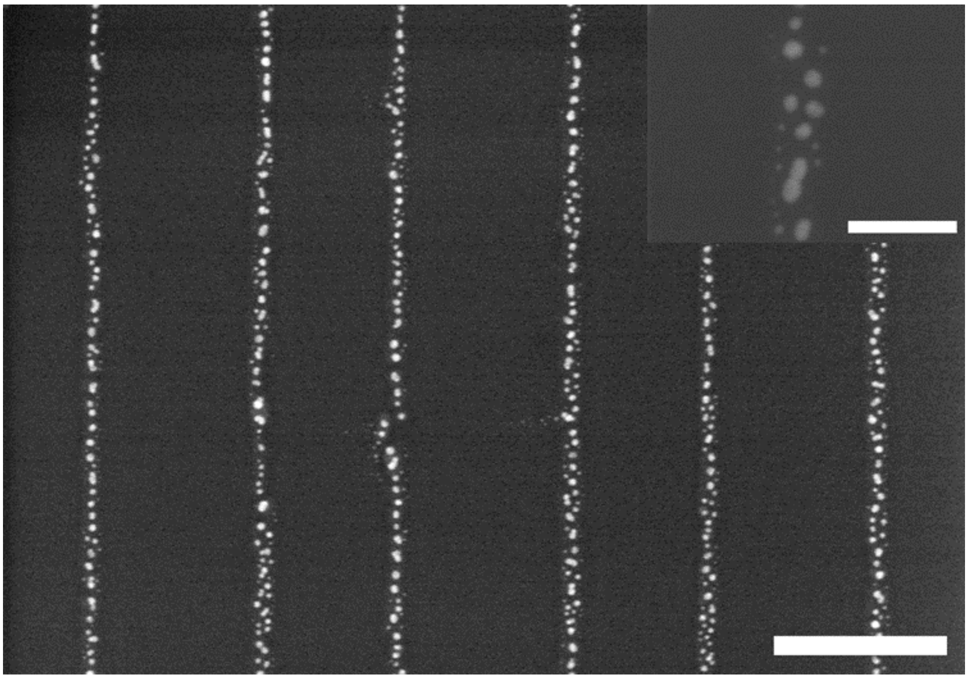

Supplementary Fig. 11. Scanning electron microscopy (SEM) images of the fabricated Ir/CNW with 500 nm scale bar. The upper inset is a high-resolution image of the sample with 50 nm scale bar.

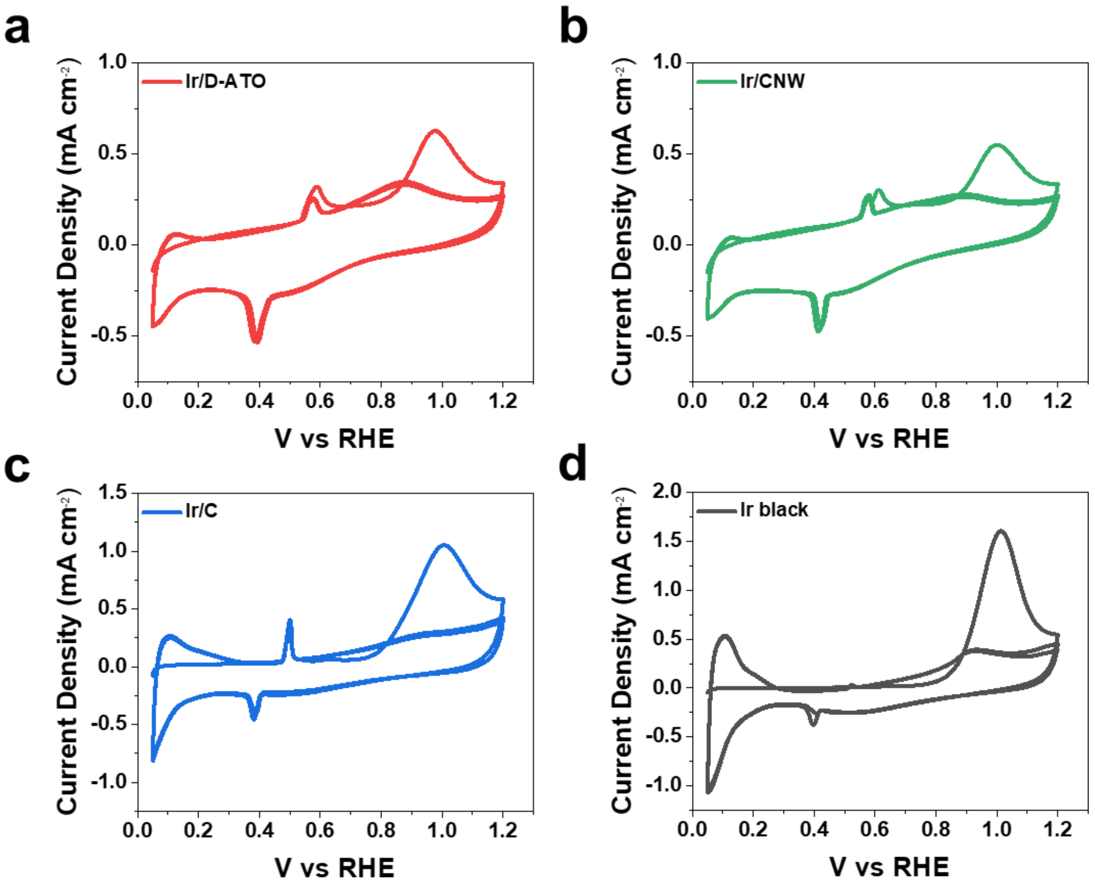

Supplementary Fig. 12. CO-stripping voltammetry cures and cyclic voltammetry curves of **a** Ir/D-ATO, **b** Ir/CNW, **c** Ir/C, and **d** Ir black to calculate the electrochemically active surface area (ECSA) estimated in 0.05M H_2SO_4.

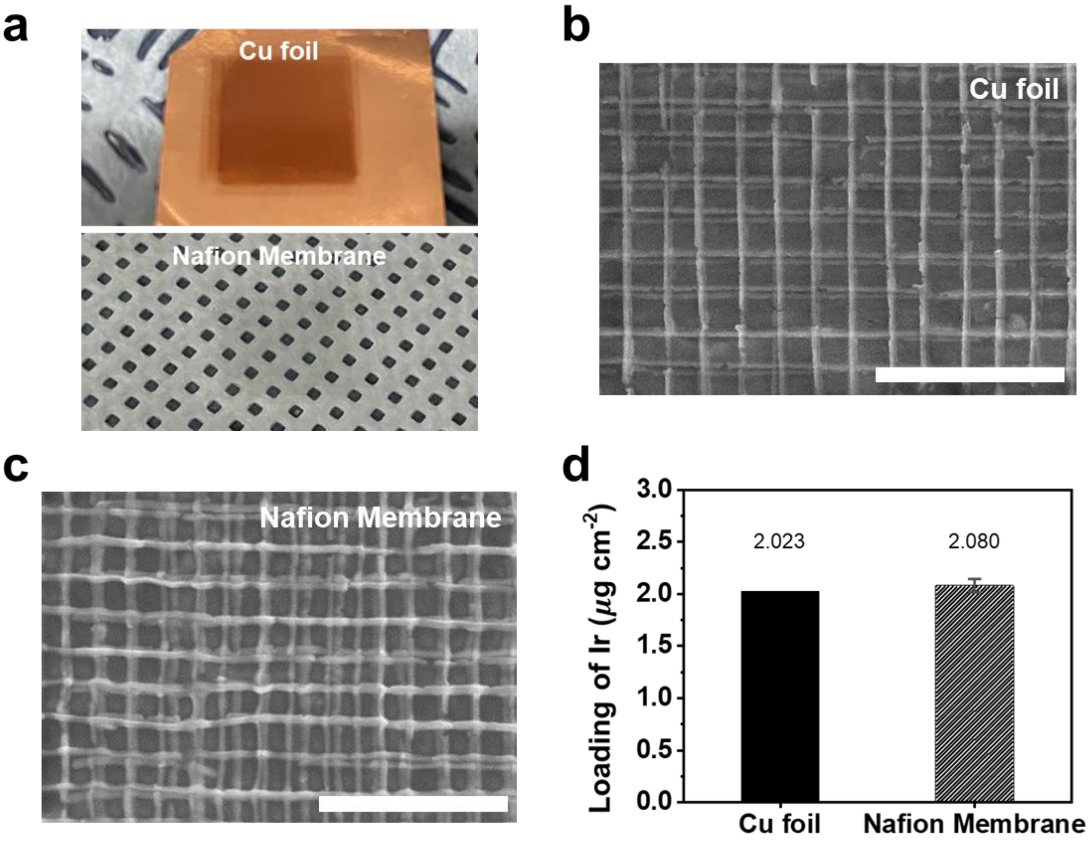

Supplementary Fig. 13. Characteristics of Ir/D-ATO before and after etching and transfer process from Cu foil to Nafion membrane. a Photographs of Ir/D-ATO fabricated on Cu foil (upper) and Nafion membrane (lower). Scanning electron microscopy (SEM) images of **b** Ir/D-ATO fabricated on Cu foil and **c** Nafion membrane with 2 μm scale bar. **d** The amount of Ir loading of Ir/D-ATO fabricated on Cu foil and Nafion membrane. The reported loading amount of Ir on the Nafion membrane represents the mean ± standard deviation (n = 3).

.

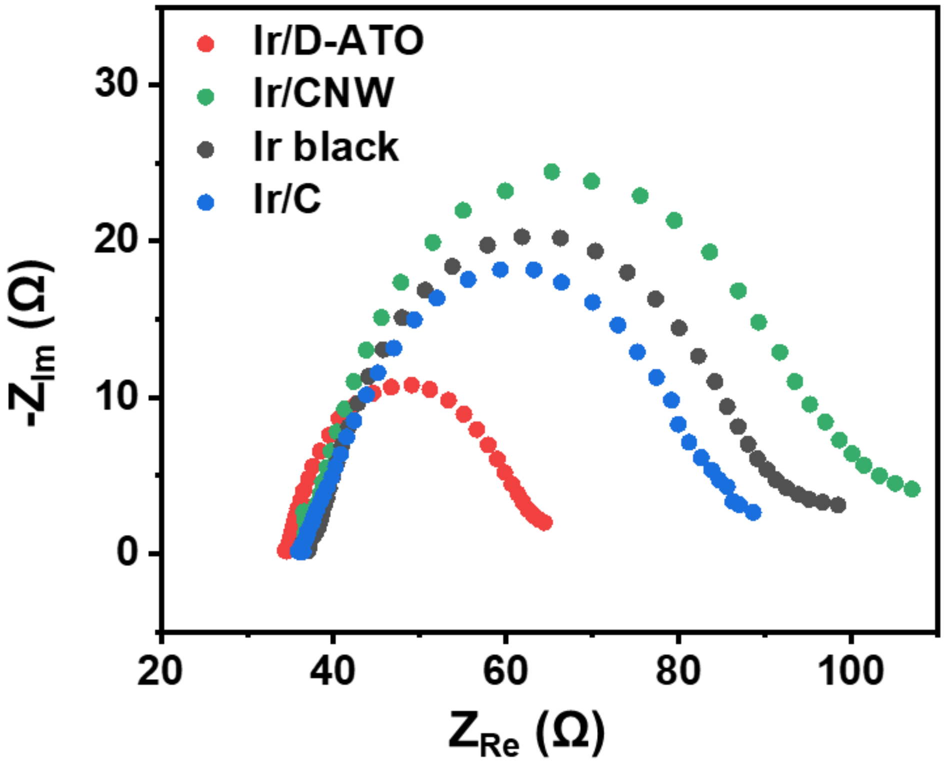

Supplementary Fig. 14. Nyquist plots for Ir/D-ATO, Ir/CNW, Ir black, and Ir/C. They were measured in 0.05M H_2SO_4 electrolyte at 1.55 V_{RHE} using electrochemical impedance spectroscopy (EIS) in a frequency range of 100000 Hz to 0.1 Hz. At 1.55 V_{RHE}, the oxygen evolution reaction occurs.

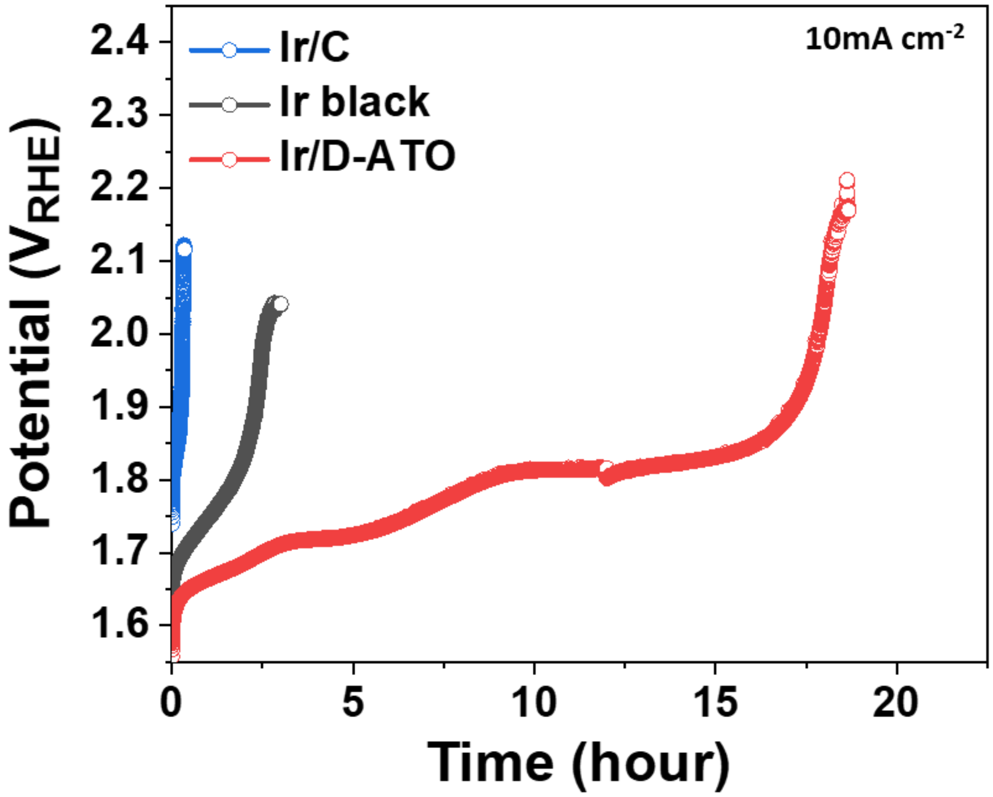

Supplementary Fig. 15. Chronopotentiometry measurements of the Ir/D-ATO and nanoparticle type commercial catalysts (Ir/C, Ir black) to estimate their stability in a half-cell at 10 mA cm^{-2}.

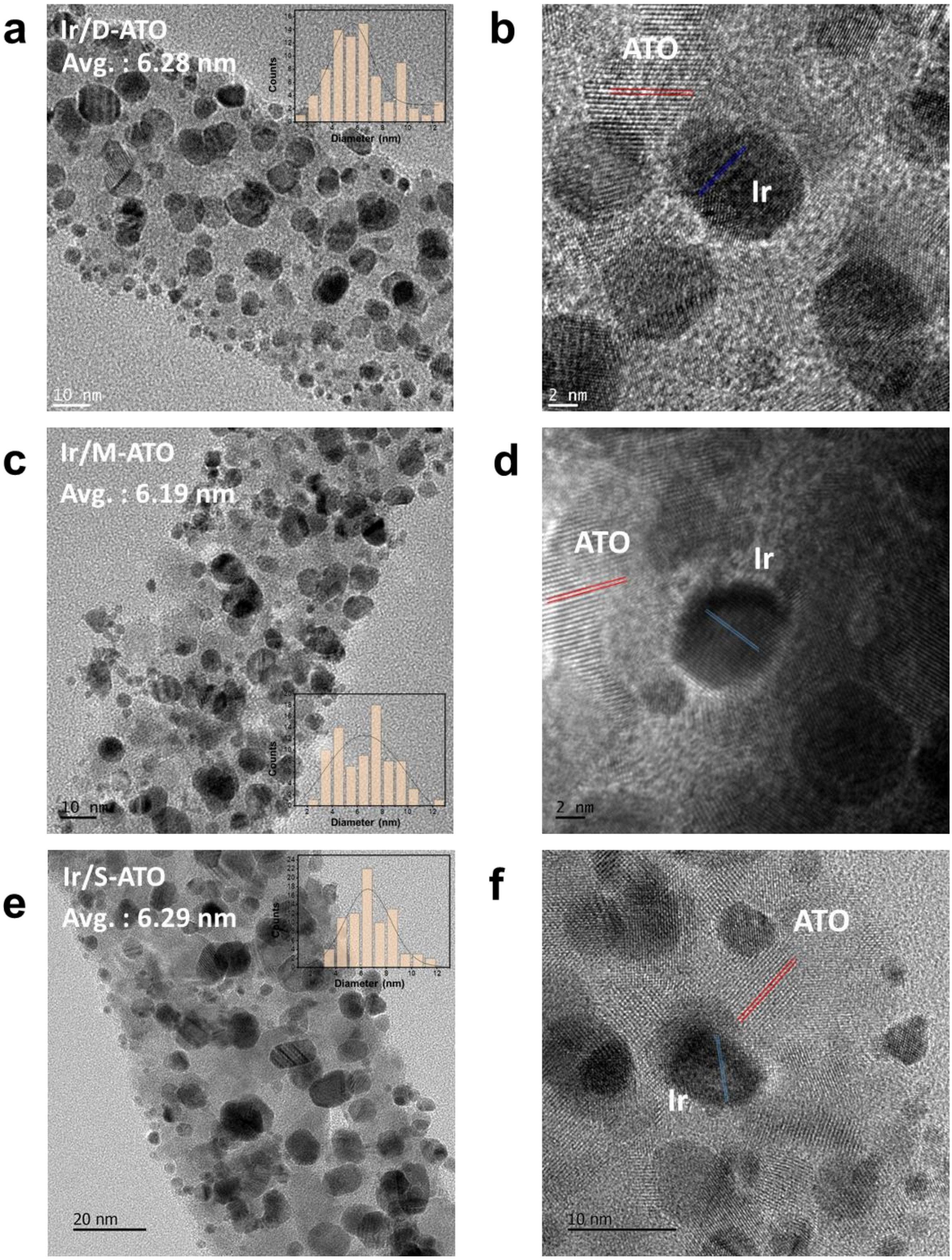

Supplementary Fig. 16. Transmission electron microscopy (TEM) and high-resolution TEM (HRTEM) images of (**a,b**) of Ir/D-ATO, (**c,d**) Ir/M-ATO, and (**e,f**) Ir/S-ATO. The (**a,c,e**) insets present particle size distribution graphs of each catalyst, which show similar average particle size.

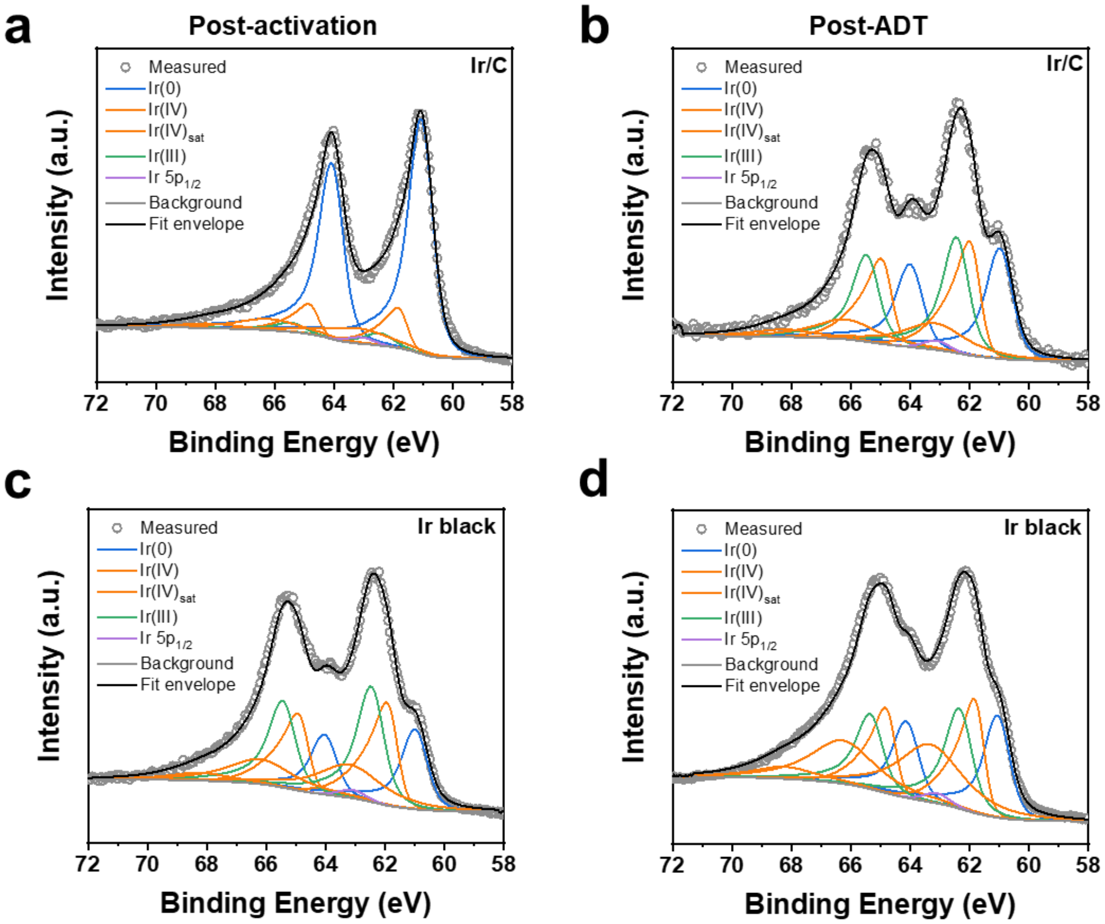

Supplementary Fig. 17. X-ray photoelectron spectroscopy (XPS) spectra of the Ir 4f level on (**a, b**) Ir/C, and (**c, d**) Ir black attained in the post-activation and post-ADT samples to compare the ratio of metallic Ir, Ir(III), and Ir(IV) of the catalysts. Note that a. u. represents arbitrary units.

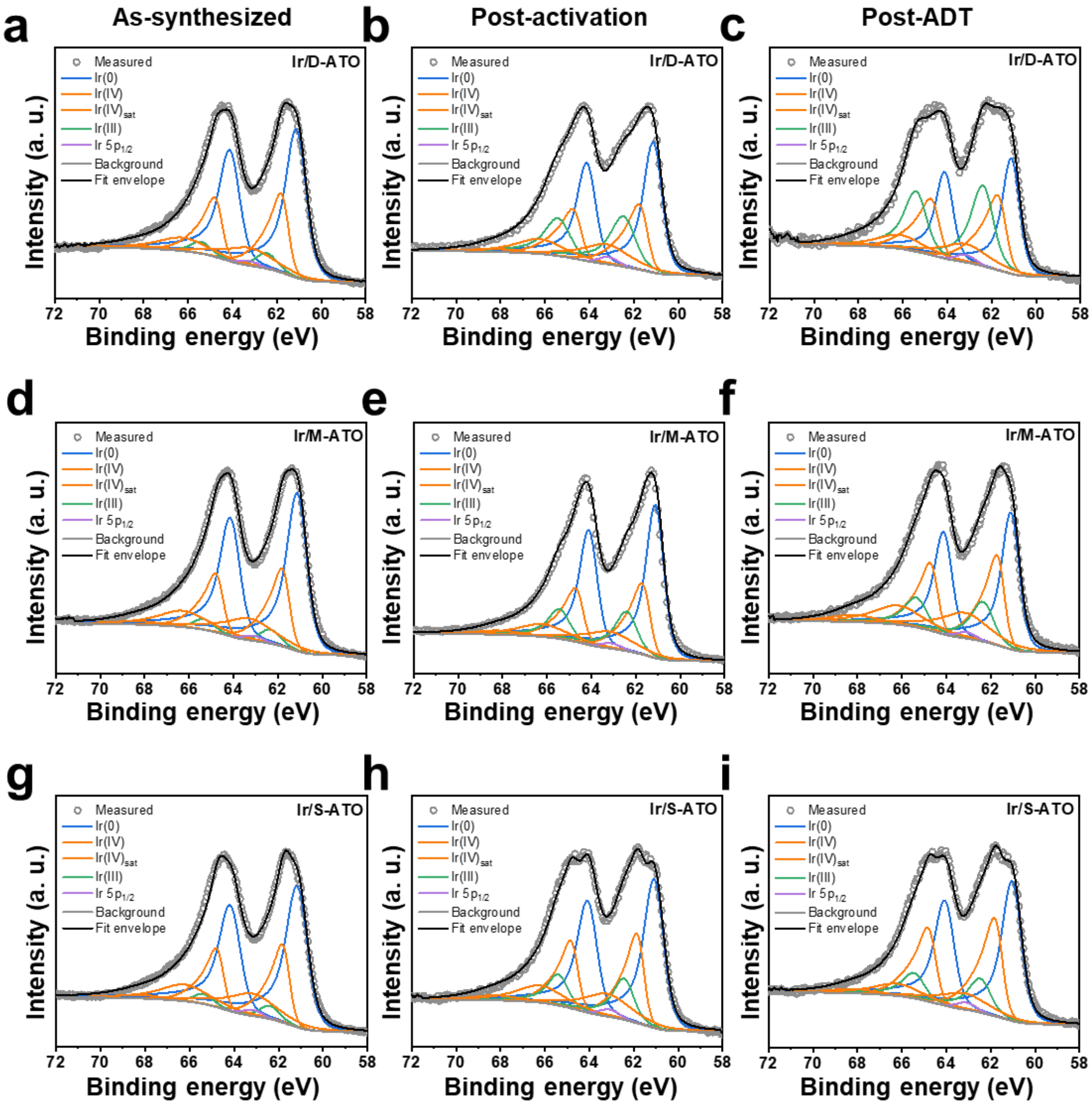

Supplementary Fig. 18. X-ray photoelectron spectroscopy (XPS) spectra of the Ir 4f level on (**a-c**) Ir/D-ATO, (**d-f**) Ir/M-ATO, and (**g-i**) Ir/S-ATO attained in the as-synthesized, post-activation, and post-ADT samples to compare the ratio of metallic Ir, Ir(III), and Ir(IV) of the catalysts. Note that a. u. represents arbitrary units.

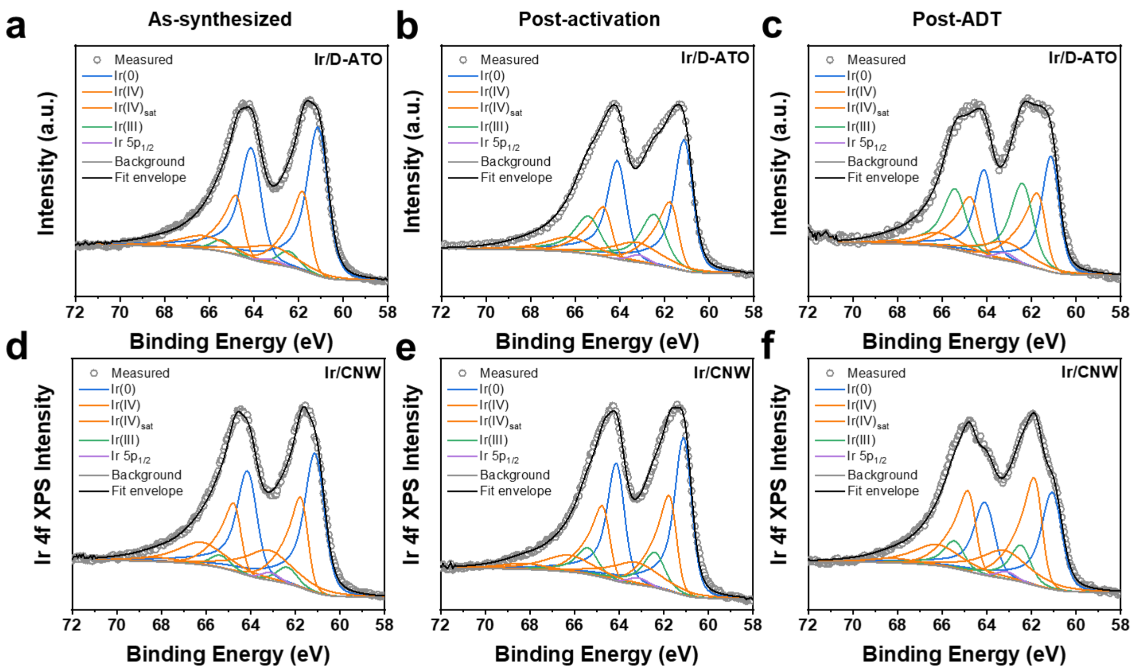

Supplementary Fig. 19. X-ray photoelectron spectroscopy (XPS) spectra of the Ir 4f level on (**a-c**) Ir/D-ATO, (**d-f**) Ir/CNW attained in the as-synthesized, post-activation, and post-ADT samples to compare the ratio of metallic Ir, Ir(III), and Ir(IV) of the catalysts. Note that a. u. represents arbitrary units.

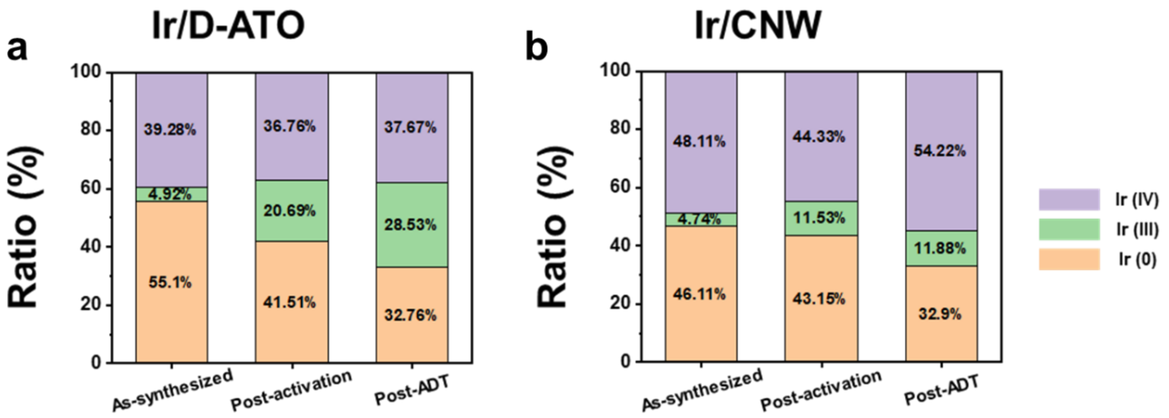

Supplementary Fig. 20. Characterization of the charge transfer capability according to the support materials. Characterization of the oxidation states of Ir in **a** Ir/D-ATO and **b** Ir/CNW for each sample (as-synthesized, post-activation, and post-ADT) by XPS.

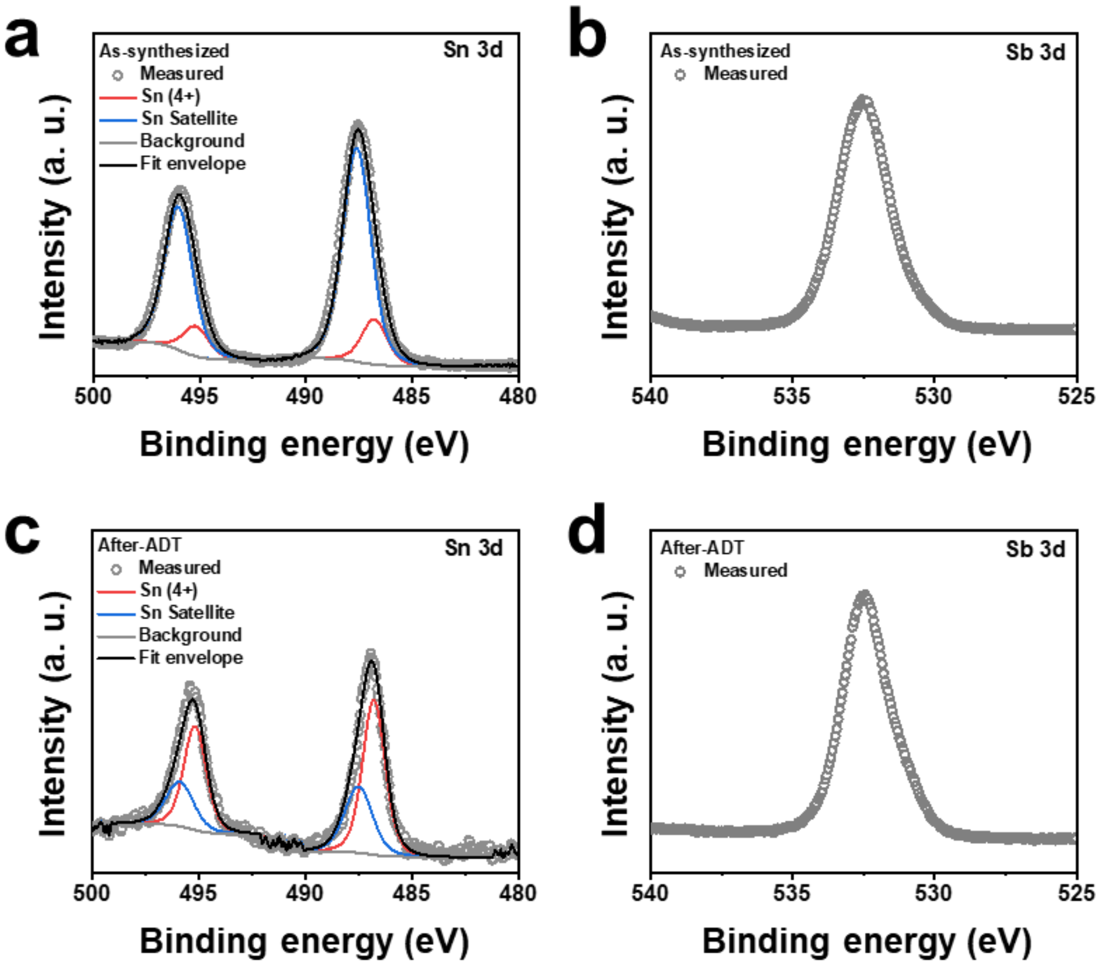

Supplementary Fig. 21. X-ray photoelectron spectroscopy (XPS) spectra of the Sn 3d and Sb 3d of Ir/D-ATO (**a, b**) before and (**c, d**) after the ADT to confirm the oxidation state changes at the support. Note that a. u. represents arbitrary units.

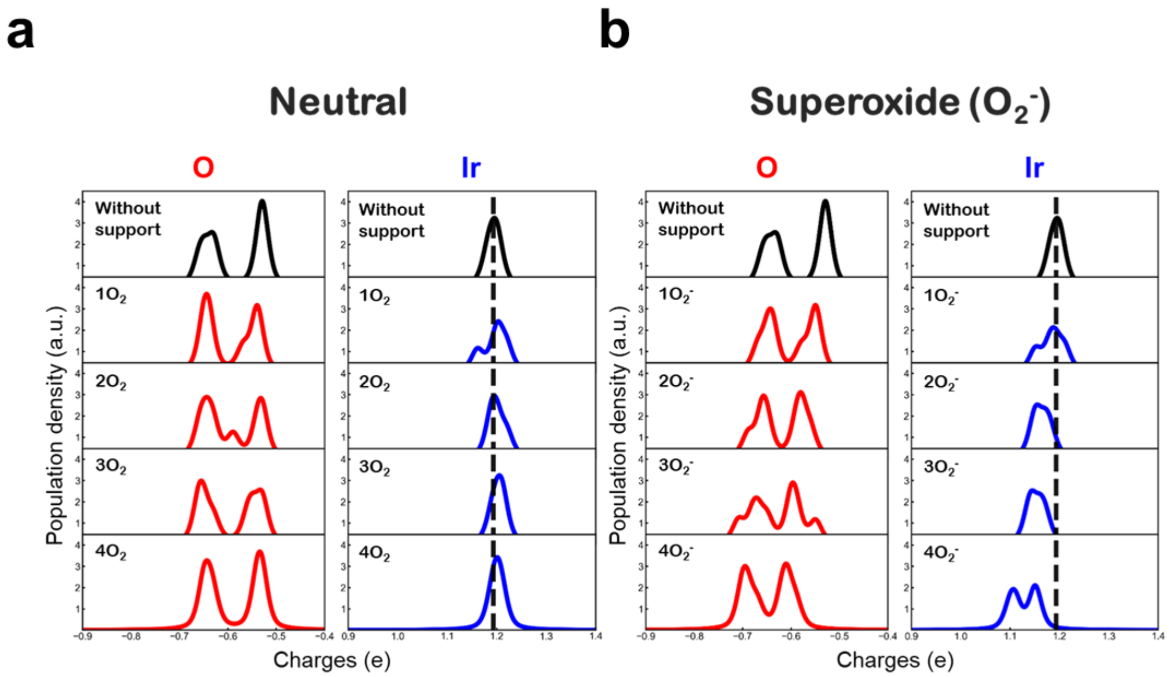

Supplementary Fig. 22. Population densities for atoms in the Ir_4O_8 cluster with varying **a** neutral O_2 and **b** charged O_2^- concentrations. Different from the superoxide, it shows that the charge state of Ir atoms hardly changes with the neutral O_2 concentration.

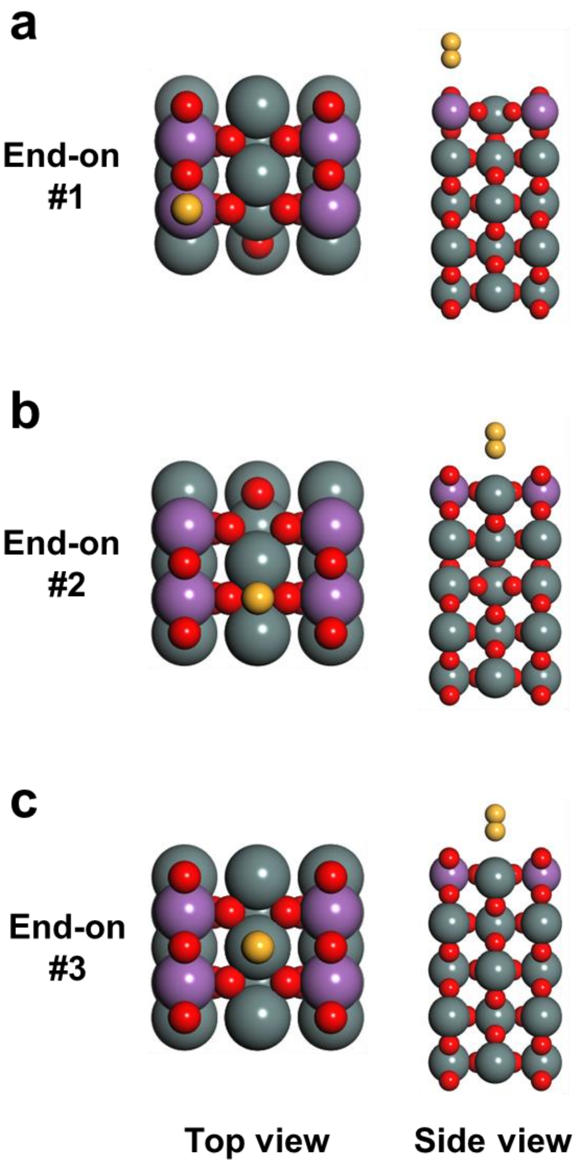

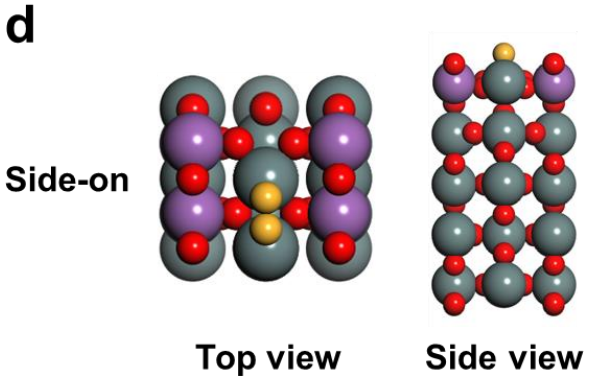

e

Adsorption mode	Charge state	Adsorption energy (eV)
**End-on #1**	Neutral	1.01
	1-	1.56
**End-on #2**	Neutral	-0.02
	1-	1.41
**End-on #3**	Neutral	-0.02
	1-	1.53
**Side-on**	Neutral	-0.71
	1-	0.46

Supplementary Fig. 23. Structures of end-on adsorption and side-on adsorption of O_2^- anion on ATO (110) surface. (**a, b, c**) Top and side views of three different end-on adsorptions of O_2^- anion **d** Top and side views of side-on adsorption of O_2^- anion **e** Adsorption energies of each adsorption mode.

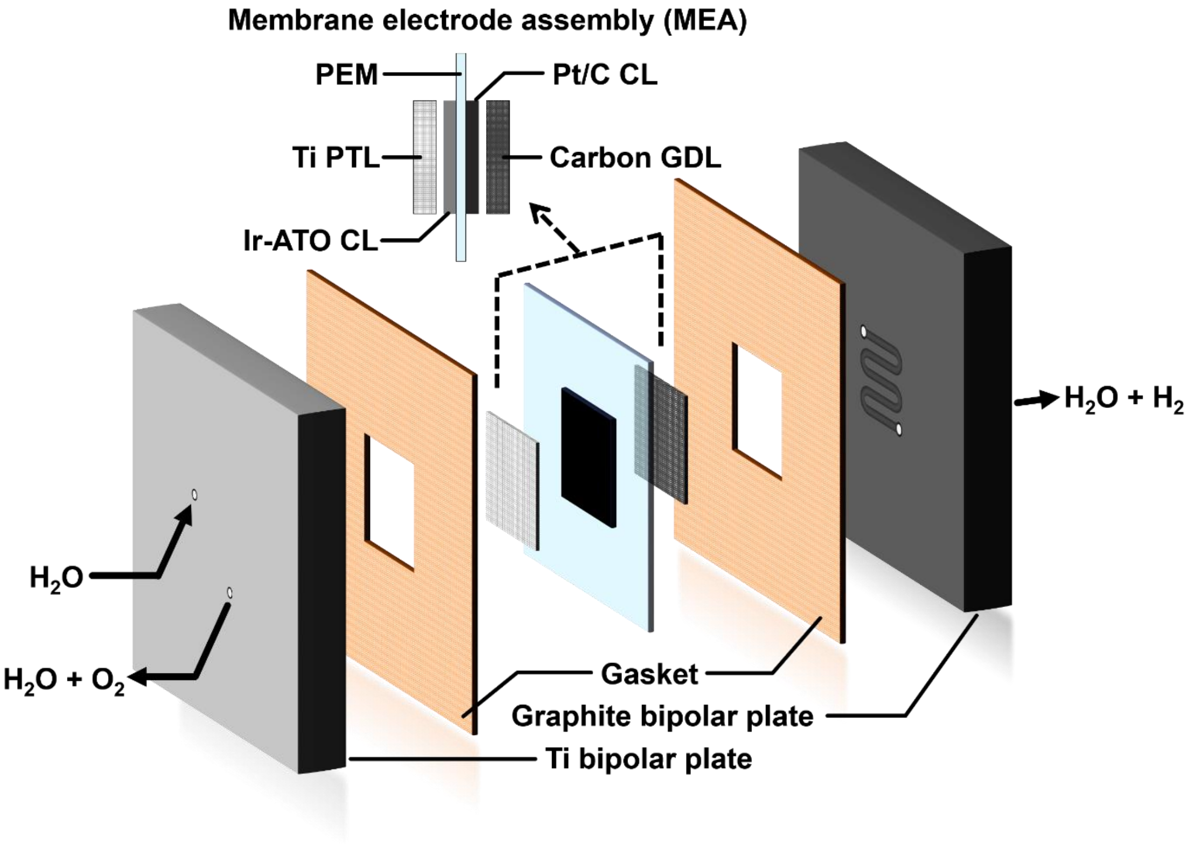

Supplementary Fig. 24. Schematic illustration of a PEMWE cell (PEM: proton exchange membrane; CL: catalyst layer; PTL: porous transport layer; GDL: gas diffusion layer).

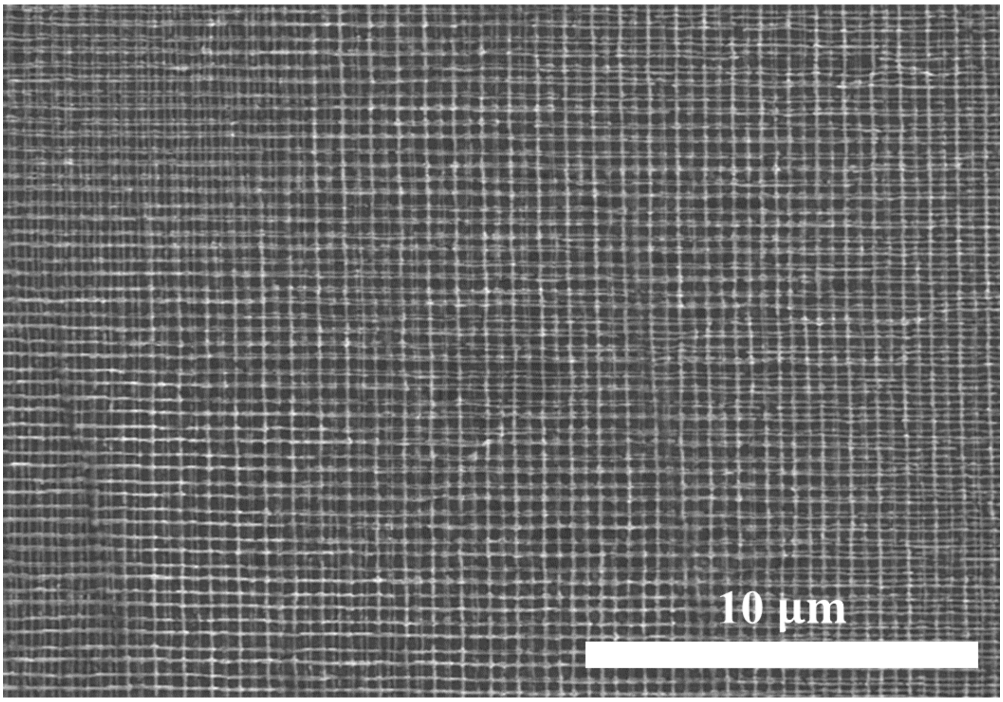

Supplementary Fig. 25. Scanning electron microscopy (SEM) image of Ir/D-ATO fabricated on Nafion membrane for single-cell measurement in PEMWE.

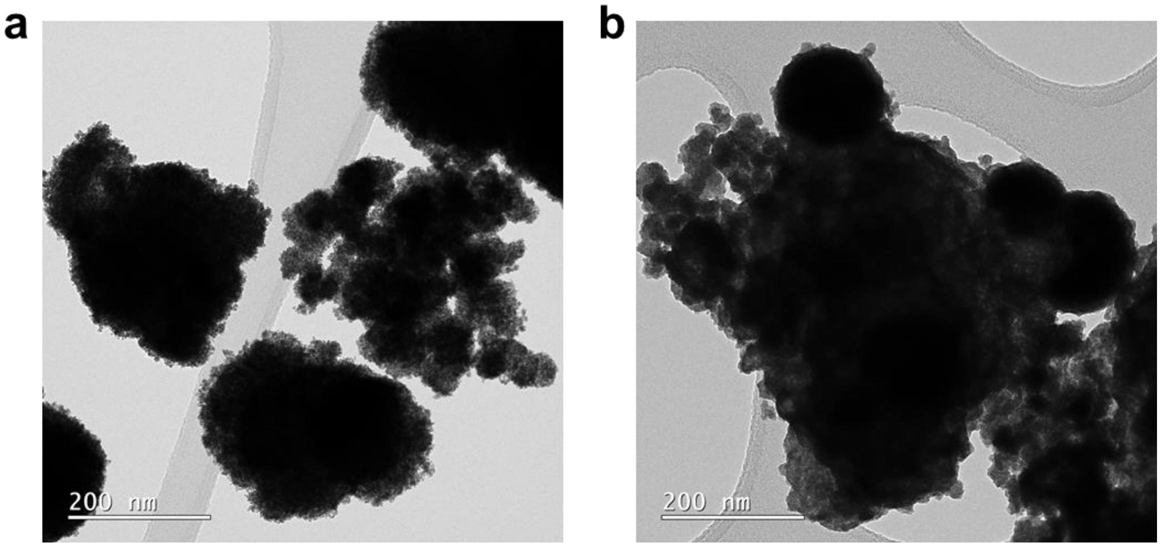

Supplementary Fig. 26. Transmission electron microscopy (TEM) images of **a** commercial Ir black and **b** Ir/TiO_2 catalyst.

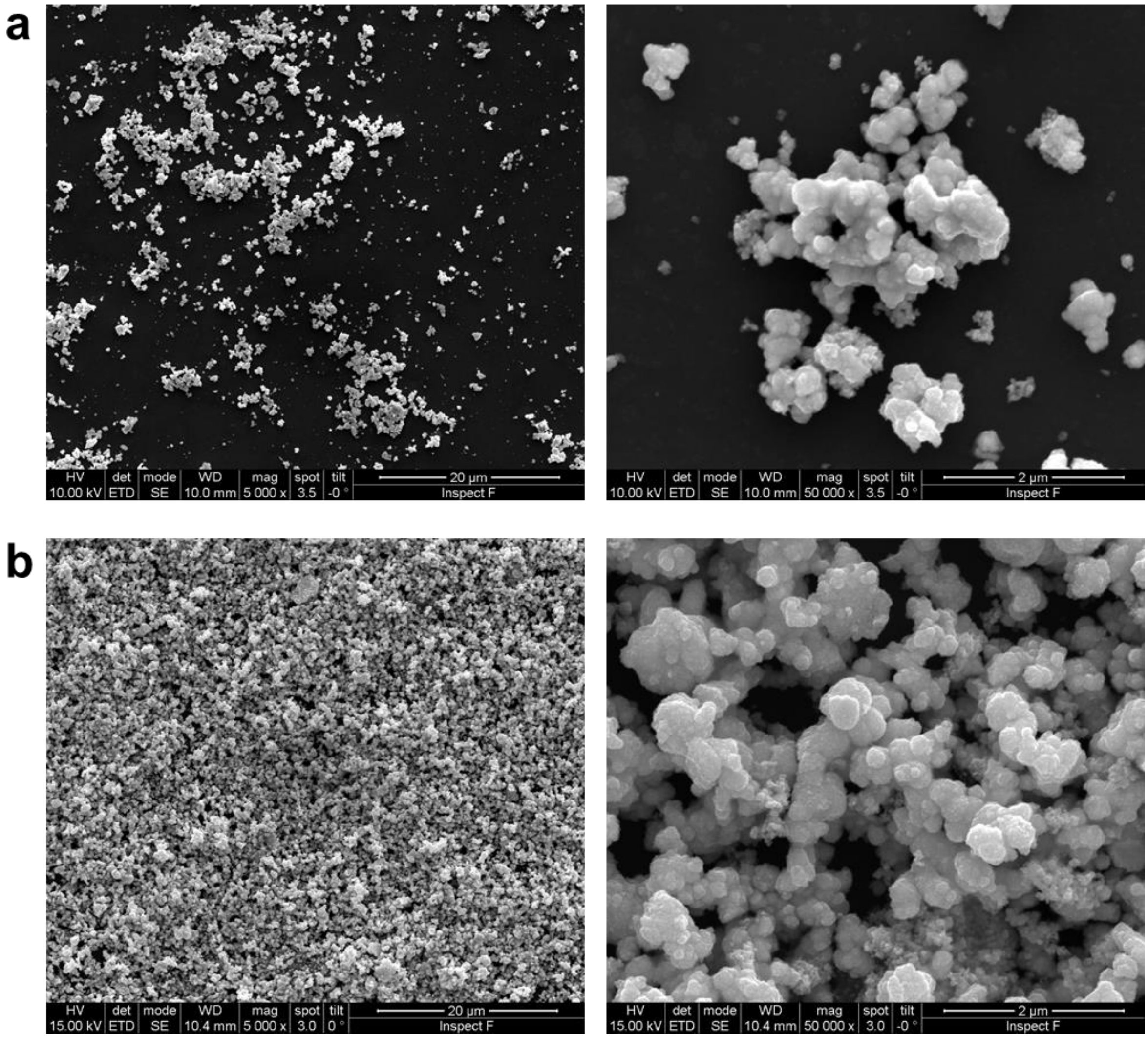

Supplementary Fig. 27. Scanning electron microscopy (SEM) images of **a** low-loaded (0.01 mg_{Ir} cm^{-2}) and **b** high-loaded (0.5 mg_{Ir} cm^{-2}) Ir black catalyst layer.

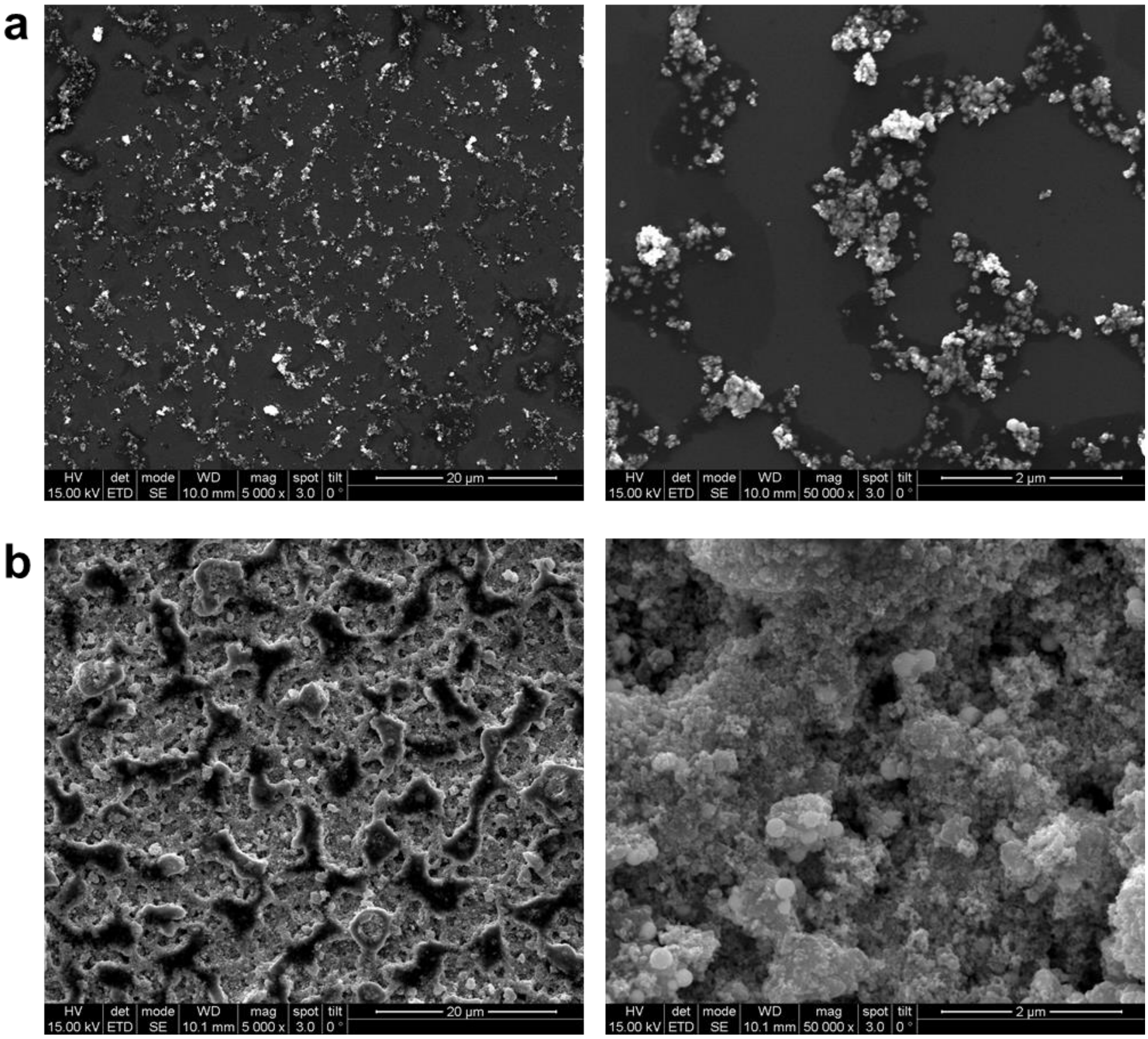

Supplementary Fig. 28. Scanning electron microscopy (SEM) images of **a** low-loaded (0.01 mg_{Ir} cm^{-2}) and **b** high-loaded (0.5 mg_{Ir} cm^{-2}) Ir/TiO_2 catalyst layer.

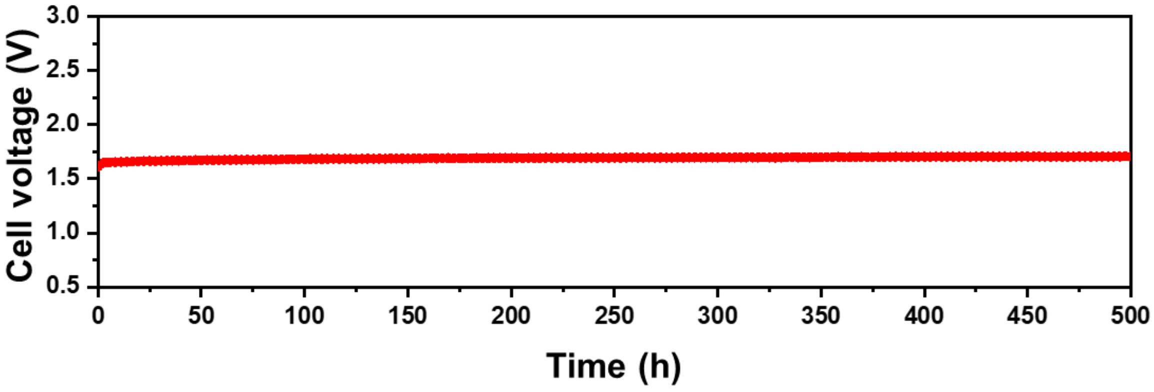

Supplementary Fig. 29. Electrochemical measurements of Ir/D-ATO in PEMWE single cell. Chronopotentiometry data for stability test of Ir/D-ATO in PEMWE single cell at 0.5 A cm^{-2}.

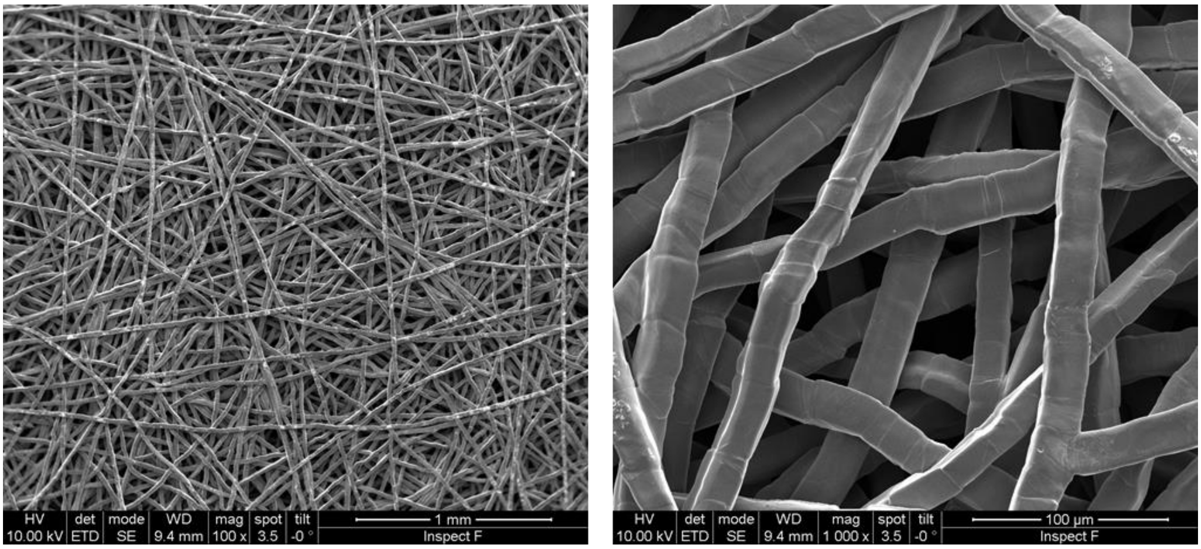

Supplementary Fig. 30. Scanning electron microscopy (SEM) images of Ti porous transport layer used in the fabrication of MEA.

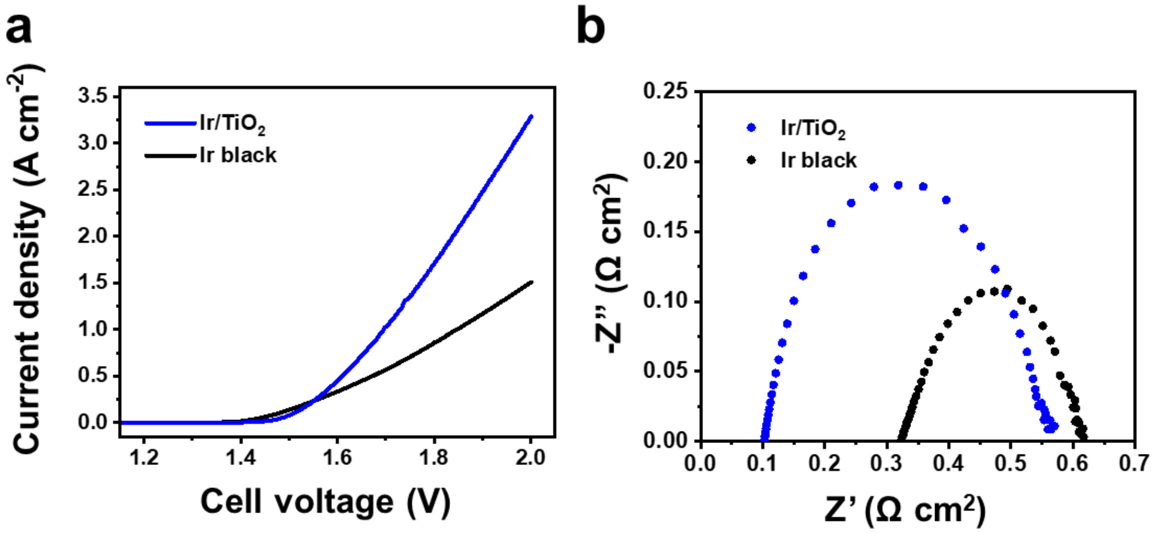

Supplementary Fig. 31. Electrochemical measurements in PEMWE single cell. a Polarization curves and **b** electrochemical impedance spectroscopy data of high-loaded Ir/TiO_2 and Ir black.

Supplementary Table 1. Measurement of loading amount of Ir for Ir/D-ATO samples and the other reference catalysts using an inductively coupled plasma mass spectrometer (ICP-MS) in a half-cell and PEMWE cell, respectively.

**Test Type**	**Sample Name**	**Loading of Ir (ug cm^{-2})**
**Half cell performance**	Ir/D-ATO	2.023
	Ir/CNW	2.023
	Ir/C	17.8
	Ir black	17.8
**PEMWE cell performance**	Ir/D-ATO	7.2
	Ir/TiO_2, Ir black	500 (high loading),<br>10 (low loading)

Supplementary Table 2. Comparison of experimental conditions (mass loading, electrolyte), mass activity, specific activity, and stability of Ir/D-ATO with previously reported Ir-based electrocatalysts using support materials in half-cell measurement.

**Catalyst**	**Mass loading (μg_{Ir} cm^{-2})**	**Electrolyte**	**Mass activity (A mg_{Ir}^{-1})**	**Specific activity (mA cm^{-2})**	**Stability**	**Ref**
Ir/D-ATO	2.023	0.05M H_2SO_4	5.975 (at 1.55 V)	4.542 (at 1.55 V)	Chronopo. 10 mA cm^{-2} 18 h	**This work**
$IrNiO_x$/meso-ATO	10.2	0.05M H_2SO_4	0.08 (at 1.51 V)	0.030 (at 1.51 V)	Chronopo. 1 mA cm^{-2} 20 h	1
Ir-ND/ATO	10.2	0.05M H_2SO_4	0.0698 (at 1.51 V)	0.029 (at 1.51 V)	Chronopo. 1 mA cm^{-2} 15 h	2
IrO_x/ATO	10.2	0.05M H_2SO_4	0.021 (at 1.51 V)	0.008 (at 1.51 V)	Chronopo. 10 mA cm^{-2} 1.5 h	3
MW-Ir/ATO	20	1M H_2SO_4	1.86 (at 1.58 V)	1.207 (at 1.58 V)	Chronopo. 10 mA cm^{-2} 15 h	4
Ir/ATO	204	0.5M H_2SO_4	0.845 (at 1.48 V)	0.571 (at 1.48 V)	N/A	5
Ir_{NP}-ITO	102	0.1M $HClO_4$	0.035 (at 1.51 V)	N/A	Chronopo. 10 mA cm^{-2} 2 h	6
IrO_2-TiO_2-245	100	0.1M $HClO_4$	0.070 (at 1.525 V)	0.028 (at 1.525 V)	R.Chronoam. 500 cycles (~90%)	7
IrNi NPNWs	25	0.1M $HClO_4$	0.732 (at 1.53 V)	0.438 (at 1.53 V)	Chronopo. 5 mA cm^{-2} 200 min	8

Supplementary References

1 Nong, H. N. *et al.* Oxide-Supported $IrNiO_x$ Core-Shell Particles as Efficient, Cost-Effective, and Stable Catalysts for Electrochemical Water Splitting. *Angew. Chem. Int. Ed.* **54**, 2975-2979 (2015).

2 Oh, H. S., Nong, H. N., Reier, T., Gliech, M. & Strasser, P. Oxide-supported Ir nanodendrites with high activity and durability for the oxygen evolution reaction in acid PEM water electrolyzers. *Chem. Sci.* **6**, 3321-3328 (2015).

3 Oh, H. S. *et al.* Electrochemical Catalyst-Support Effects and Their Stabilizing Role for IrO_x Nanoparticle Catalysts during the Oxygen Evolution Reaction. *J. Am. Chem. Soc.* **138**, 12552-12563 (2016).

4 Massue, C. *et al.* High-Performance Supported Iridium Oxohydroxide Water Oxidation Electrocatalysts. *Chemsuschem* **10**, 1943-1957 (2017).

5 Karimi, F. & Peppley, B. A. Metal Carbide and Oxide Supports for Iridium-Based Oxygen Evolution Reaction Electrocatalysts for Polymer-Electrolyte-Membrane Water Electrolysis. *Electrochim. Acta* **246**, 654-670 (2017).

6 Lebedev, D. & Coperet, C. Small, Narrowly Distributed Iridium Nanoparticles Supported on Indium Tin Oxide for Efficient Anodic Water Oxidation. *ACS Appl. Energy Mater.* **2**, 196-200 (2019).

7 Oakton, E. *et al.* IrO2-TiO2: A High-Surface-Area, Active, and Stable Electrocatalyst for the Oxygen Evolution Reaction. *ACS Catal.* **7**, 2346-2352 (2017).

8 Wang, Y. *et al.* Nanoporous Iridium-Based Alloy Nanowires as Highly Efficient Electrocatalysts Toward Acidic Oxygen Evolution Reaction. *ACS Appl. Mater. Inter.* **11**, 39728-39736 (2019).
